# Takotsubo Cardiomyopathy—Acute Cardiac Dysfunction Associated With Neurological and Psychiatric Disorders

**DOI:** 10.3389/fneur.2019.00917

**Published:** 2019-08-22

**Authors:** Sylvia J. Buchmann, Dana Lehmann, Christin E. Stevens

**Affiliations:** ^1^Charité Universitätsmedizin Berlin, Corporate Member of Freie Universität Berlin, Humboldt-Universität zu Berlin, and Berlin Institute of Health, Berlin, Germany; ^2^Department of Neurology, Augustahospital Anholt, Isselburg-Anholt, Germany

**Keywords:** Takotsubo (stress) cardiomyopathy, autonomic (vegetative) nervous system, psychiatric disorders, neurological disorders, affective disorders

## Abstract

Takotsubo cardiomyopathy (TTC) is an acute and reversible cardiac wall motion abnormality of the left myocardium. Although many studies focused on etiology, diagnostic and treatment of TTC, precise clinical guidelines on TTC are not available. Research revealed emotional and physical triggering factors of TTC and emphasized the association of TTC with psychiatric and particularly acute neurological disorders. Similar clinical presentation of acute coronary syndrome (ACS) and TTC patients, makes an anamnestic screening for TTC risk factors necessary. In psychiatric anamnesis affective disorders and chronic anxiety disorders are presumably for TTC. Subarachnoid hemorrhages and status epilepticus are typical acute neurological associated with a higher risk for TTC. Moreover, magnetic resonance imaging (MRI) studies reveled brain alterations of the limbic system and reduced connectivity of central autonomic nervous system structures. Diagnosis of TTC is made by elevation of cardiac enzymes, electrocardiogram (ECG) and visualization of myocardial wall motion. Major differential diagnoses like acute coronary syndrome and myocarditis are hereby in synopsis with anamnesis with respect of possible emotional and physical triggering factors of TTC ruled out. In most cases the TTC typical wall motion abnormalities resolve in weeks and therapy is only necessary in hemodynamic instable patients and if rare complications, like cardiac wall ruptures occur. Recently, the two-parted International expert consensus document on Takotsubo syndrome was published, providing a detailed characterization of TTC and allows clinicians to understand this cardiac dysfunction with a multidisciplinary view.

## Introduction

Takotsubo cardiomyopathy (TTC), also known as left ventricular apical ballooning syndrome (LVBS), transient apical ballooning, stress cardiomyopathy, or broken heart syndrome, is an acute and reversible wall motion abnormality classically of the left ventricular myocardium and was firstly described by Sato et al. (1) and Ghadri et al. (2). The Japanese term “Takotsubo” means “octopus pot” and describes the characteristic left ventricular end-systolic apical ballooning phenomenon, which can be visualized in transthoracic echocardiogram (TTE) or coronary angiography with left ventriculography. The classical morphological pattern of TTC is an apical akinesia with basal hyperkinesia of the left ventricular myocardium (3). However, there have been further wall motion abnormalities in TTC described, such as basal, midventricular, and lateral akinesia of the left ventricular myocardium and also involvement of the right ventricular myocardium as part of a biventricular involvement or isolated right ventricular wall motion abnormality (3). Typically, TTC patients present with clinical symptoms suggestive for an acute coronary syndrome (ACS), such as chest pain, dyspnea, syncope, and nausea with sudden onset after an emotional or physical stressor (4). From an epidemiological point of view patients diagnosed with TTC are typically postmenopausal female patients with a mean age of 66.8 years (5, 6). However, the epidemiology of TTC was shown to be more complex according to various retrospective studies. Biomarkers used in diagnosis of TTC are the cardiac enzymes troponin, creatine kinase, and N-terminal prohormone of brain natriuretic peptide (NT-proBNP), which are classically elevated, but show lower peak values than in patients with ACS. Although, ECG changes in TTC patients are not specific, most commonly ST-elevations in leads II, III, aVF, aVR, and V5 to V6 are seen (7). Additionally, repolarization abnormalities (T-wave inversions) are commonly seen in ECGs of TTC patients, as well as QTc-prolongations. Recently, the International Takotsubo Diagnostic Criteria (InterTAK Diagnostic Criteria) as part of an international expert consensus document have been published and support differentiation of TTC patients with no ST-elevation in ECG and ACS patients (8). The clinical challenge in emergency rooms is to rule out an ACS as most important differential diagnosis of TTC. Moreover, acute infectious myocarditis or pericarditis are relevant differential diagnoses of TTC. Complications of TTC include ventricular arrhythmias, acute heart failure with cardiogenic shock as a result of primary pump failure or left ventricular outlet tract obstruction, whereas rare complications are cardiac wall ruptures or formation of left ventricular thrombus (2, 9). Generally, the wall-motion abnormalities normalize within hours to weeks in TTC patients (9). We reviewed the current available literature to outline the pathophysiological mechanisms of TTC, focusing on linking TTC to psychiatric, and neurological disorders. Moreover, we briefly describe the diagnostical workflow in emergency rooms of patients with suggested TTC. We conclude our review with a concise overview about therapeutic strategies of TTC.

## Epidemiology of Takotsubo Cardiomyopathy

The incidence and prevalence of TTC are reported to be increasing, certainly due to a more sensitive clinical screening of patients in e.g., chest pain units for TTC. Deshmukh et al. studied the occurrence of TTC from the Nationwide Inpatient Sample database of US hospitalizations based on the International Classification of Diseases (ICD) in 2008 and demonstrated that 0.02% of all patients hospitalized in the US were diagnosed with TTC (10). Two percent of patients with clinical suspected ACS were diagnosed with TTC (7). Interestingly, data derived from the International Takotsubo Registry revealed patients characteristics with TTC in the United States and Europe and showed that of 1,750 studied patients diagnosed with TTC 89.8% were postmenopausal women with a mean age of 66.8 years (5, 6). Importantly, higher in-hospital mortality rates of male TTC patients compared to female TTC patients have been observed retrospectively (11, 12). However, Patel et al. found in their analysis no significant sex difference in respect of overall mortality rates of TTC patients aged ≥50 years (11). Remarkably, a significant higher prevalence of neurologic or psychiatric disorder rates among TTC patients compared to ACS patients has been reported (5). Additionally, male TTC patients ≥50 years showed physical triggers prior to the onset of TTC more often, whereas female TTC patients ≥50 years seem to suffer from premorbid psychiatric disorders more frequently (11). Notably, female patients showed higher recurrence rates of TTC compared to male TTC patients (11). Singh et al. detected an annual rate of TTC recurrence of 1.5% (13). Moreover, one retrospective analysis revealed a TTC recurrence of 6.1% during a follow up period of 6 years (14). Effectiveness of pharmacologic therapy in order to prevent reoccurrence of TTC is under current investigation. Tendentially, prescription of ACE-inhibitors is reported to be inversely correlated to recurrence of TTC in retrospective analysis (15). Furthermore, β2-adrenergic agonist agents intake was found to be associated with higher TTC prevalence (16).

## Pathophysiological Mechanisms of Takotsubo Cardiomyopathy

The underlying pathophysiological mechanism of TTC is not completely understood until today. Over the last decades numerous animal experiments and clinical studies have been conducted to elucidate the pathophysiology of TTC, outlining TTC as a multifactorial acute, and reversible cardiac disorder. Nevertheless, it is unquestioned that emotional and physical stress are frequent triggers of TTC (17). Initially, Sato et al. explained the pathophysiology of TTC with simultaneous spasms of coronary arteries (1, 18). The theory of simultaneous coronary vasospasm as underlying mechanism of TTC was disproved, as endomyocardial biopsies taken from TTC patients showed histopathological patterns of myocardial abnormalities, which are not characteristic for infarcted, stunned or hibernating myocardium (7). Furthermore, coronary microvascular dysfunction as etiology of TTC has been studied, but data are still not distinct up to now (7, 9).

**Figure 1:**
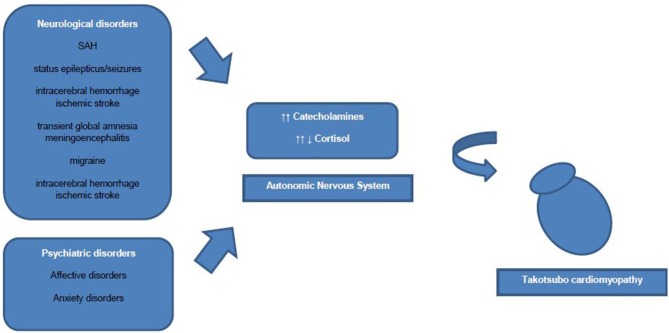
Linking neurological and psychiatric disorders to Takotsubo cardiomyopathy.

However, an association of increased sympathetic activity resulting in systemic blood catecholamine excess, and TTC has been demonstrated in numerous studies (2). Some authors discuss increased blood catecholamine levels rather as a triggering factor than an underlying pathophysiological mechanism of TTC. Interestingly, to date only one study showed extremely high plasma concentrations of catecholamines, whereas other studies showed nearly normal catecholamine blood levels in TTC patients (9). Research has drawn attention to the role of β2-adrenoceptors, as high epinephrine blood levels induce a β2-adrenoceptor coupling change from membranous Gs proteins to Gi proteins with a consecutive negative inotropic effect (19). Therefore, the reversible nature of ventricular ballooning after normalization of catecholamine blood levels could be explained by these compensatory biochemical processes. Additionally, regional differences in myocardial expression of β2-adrenergic receptor density have been shown, which mediate the cellular effects of the increased catecholamine blood concentrations and explain the regional left ventricular myocardial stunning (2). Besides circulating blood catecholamines, secreted from the adrenal medulla, ventricular sympathetic nerve fiber terminals release norepinephrine and a hyperactivation of these cardiac sympathetic nerve terminals with increased synaptic norepinephrine levels and consecutive activation of post-synaptic α_1_-, β_1_-, and β_2_–receptors as leading pathomechanism is discussed currently. However, sympathetic nerve fiber density is higher in basal myocardium as in ventricular myocardium and therefore blood circulating epinephrine seems to have a greater influence on apical ventricular myocardium then norepinephrine released from the sympathetic nerve terminals in apical myocardium (19) (Figure 1).

Recent research on the pathogenesis of TTC demonstrated an association of inflammatory myocardial processes in TTC patients, linking catecholamine stress-induced TTC to inflammatory responses of the myocardium in experimental animal studies. Wilson et al. characterized the myocardial inflammatory response in TTC based on animal experimental studies with catecholamine induced TTC, showing a predominant myocardial M1 macrophages infiltration in TTC without a switch of M1 macrophages (proinflammatory tissue destructive) to M2 macrophages (anti-inflammatory tissue reparative/profibrotic) (20). Importantly, Wilson et al. found in their study a positive correlation of increasing EF with the percentage of M2 macrophages (20). However, it remains to be elucidated, whether inflammatory myocardial processes are occurring prior TTC or are results of TTC in further animal-based experimental and clinical studies, in order to develop specific therapeutic strategies. Not only local myocardial inflammatory processes have been described, but also persistent systemic peripheral inflammatory response in TTC patients has been studied, making potential long term pharmacological anti-inflammatory treatment of TTC considerable. Systemic peripheral inflammation denoted in elevated serum levels of interleukin-6, chemokine ligand 1, CD14^++^CD16^−^ monocytes, and non-classical CD14^+^CD16^++^ monocytes have been described in clinical studies (21). Whereas, serum levels of intermediate CD14^++^CD16^+^ monocytes and non-classical CD14^+^CD16^++^ monocytes were reduced (21). Scally et al. reported persistent peripheral systemic inflammation processes in TTC patients in follow-up measurements of pro-inflammatory cytokines, whereby serum concretions of interleukin 6 and interleukin 8 remained elevated (21). In clinical settings inflammatory processes in context of TTC can be visualized as myocardial edema in cardiac magnetic resonance imaging (MRI) more favorable in T2 weighted imaging of the myocardium (21, 22).

Recent studies focus on cardiac ion channel activity modulated by inflammatory cytokines and the resulting change of cardiac action potential duration, which should be mentioned as an association of inflammation and TTC has been previously described. It has been postulated, that both circulating cytokines directly affect ion channels of cardiomyocytes and indirectly increase the risk for the occurrence of cardiac electrophysiological disturbance through increased sympathetic output from central and peripheral autonomic nervous system nerve fibers (23). These changes in cardiac action potentials are mainly pictured through QTc prolongation in ECG and has been proven to be associated with high blood levels of acute phase proteins (23). An increase of QT interval is therefore called an acquired cardiac channelopathy. The suggested pathophysiological mechanisms are both changes in expression and function of potassium and calcium channels (23). It has been demonstrated, that IL-1β and IL-6 enhance cardiac calcium channels (23). Contrary, potassium channels have been reported to be reduced expressed via activation of TNFα pathways (23). Interestingly, so far no studies demonstrated a change of expression or function of cardiac sodium channels via cytokines (23). Moreover, an inflammatory reflex has been described in patients with QT prolongation. The underlying cardiac changes are mediated through a cytokine mediated central activation of sympathetic nerve fibers. As a consequence of this activation cytokine production and activation of β2-adrenergic receptors expressed in circulating lymphocytes and monocytes is decreased (23). The sympathetic nerve fiber activation results in cardiac activation of calcium and potassium channels leading to increased duration of cardiac action potential (23). Increased calcium ion influx and decreased potassium efflux from cardiomyocytes results in an increase of cardiac action potential and therefore a prolongation of the QT interval (24). Additionally, studies have shown an strong association of high c-reactive protein blood levels and QT interval prolongation (24). Not only acute phase proteins have been demonstrated to modulate cardiac ion channel function, but also antibodies, e.g., anti-Sjögren's-syndrome-related antigen A have been found to influence potassium channels of ventricular cardiomyocytes resulting in QT prolongation (25).

Numerous studies suggested the existence of genetic predisposition for TTC. Genetic polymorphisms for cardiac α1-, β1-, and β2-adrenergic receptors, GRK5, and estrogen receptors have been described (3). However, the genetic associates need to be evaluated in larger TTC cohorts in the future.

## Takotsubo Cardiomyopathy and Neurological Disorders

Multiple clinical cases of emerging TTC after acute disorders of the central nervous system published. Over the last decades remarkable interactions of the brain and heart derived from clinical complications of patients with neurological disorders followed or accompanied by newly cardiac disorders have been described. Hence, the term brain-heart-syndrome was introduced compromising cardiac damage following brain disorders (26). Table 1 summarizes the key studies of the association of TTC and neurological and psychiatric disorders.

**Table 1:** Key studies on association between Takotsubo cardiomyopathy with neurological and psychiatric disorders.

**Study**	**Study type**	**Number of patients**	**Associated neurological psychiatric disorder**
Morris et al. (27)	Cross-sectional retrospective analysis	National inpatient sample TTC with acute neurological disorder = 155,105 TTC without acute neurological disorder = 149,273	Association of TTC with following acute neurological disorders: SAH (OR 11.7; 95% CI 10.2–13.4), status epilepticus (OR 4.9; 95% CI 3.7–6.3), seizures (OR 1.3; 95% CI 1.1–1.5), transient global amnesia (OR 2.3; 95% CI 1.5–3.6), meningoencephalitis (OR 2.1; 95% CI 1.7–2.5), migraine (OR 1.7; 95% CI 1.5–1.8), intracerebral hemorrhage (OR 1.3; 95% CI 1.1–1.5), and ischemic stroke (OR 1.2; 95% CI 1.1–1.3. Traumatic brain injury is negative associated with TTC (OR 0.7; 95% CI 0.6–0.9)
Lee et al. (28)	Cross-sectional retrospective analysis	Mayo Clinic neurological intensive care unit SAH-induced TTC = 8 No controls	Association of TTC with aneurysmal SAH with following cerebral vasospasm (*n = 6*) and pulmonary edema (*n* = 5)
Templin et al. (5)	Cross-sectional retrospective analysis	International Takotsubo registry TTC = 455 ACS = 455	55.8% of TTC patients had history or an acute episode of neurologic or psychiatric disorder, whereas only 25.7% ACS patients had neurological psychiatric disorder (*P* < 0.001)

*ACS, acute coronary syndrome; SAH, subarachnoid hemorrhage; TTC, Takotsubo cardiomyopathy*.

Common acute neurological disorders associated with the occurrence of TTC are ischemic strokes, subarachnoid hemorrhages and seizures (2). Whereas, subarachnoid hemorrhages were found to be strongly associated with TTC in various studies. In a recently published cross-sectional study the strongest associations between acute neurological diseases with following TTC have been found for subarachnoid hemorrhages, status epilepticus and less commonly for seizures (27). Interestingly, Morris et al reported a negative association of traumatic brain injury and TTC (27). Further neurological disorders associated with TTC are transient global amnesia, meningoencephalitis, migraine headache, intracerebral hemorrhage and ischemic stroke (27). In one study patients with aneurysmal subarachnoid hemorrhage induced TTC showed a high association with inter alia (i.a.) following cerebral vasospasm, pulmonary edema and longer duration of intubation (28). Hence, acute neurological disorders are counted to be an important physical trigger of TTC and every patient with symptoms suggestive for ACS should be worked up carefully regarding possible TTC. Over the last decades research focused on the hypothalamic-pituitary-adrenal axis (HPA-axis) as major neuroendocrine system regulating the release of i.a. cortisol from the adrenal gland, shifting the metabolism to higher stress levels (26). Higher serum cortisol levels have been correlated with stroke severity and insular damage (26). Additionally, the sympathetic activity levels are increased in patients with ischemic stroke due to activation of the HPA axis, resulting in i.a. significant increases of catecholamine blood levels. Those lead to higher risks of occurrence of arrhythmias and myocardial damage with resulting inflammatory responses of the affected myocardial area (26, 29). Local myocardial necrosis can lead to advanced inflammatory processes with antigen-dependent autoimmunity and exaggerated immune-mediated tissue damage, which needs to be further investigated in TTC patients (29). Furthermore, animal studies have shown an increase of plasma catecholamine levels after ischemic stroke, which is directly proportional to the incidence of myocardial damage followed by cardiac damage (26). Especially, ischaemic or hemorrhage stroke of the insular cortex are reported to have major influence on cardiac function (26). Interestingly, the right hemisphere seems to control the sympathetic activity, whereas the left hemisphere regulates parasympathetic activity (26). For example infarctions of the left hemisphere of the brain are associated with arrhythmias, a decreased cardiac wall motion and an increased risk of adverse cardiac outcome (26).

Moreover, anatomical brain alterations have been described in TTC patients. A MRI study performed with a TTC cohort derived from the International Takotsubo Registry visualized reduced gray-matter volume of structures in the brain areas of the limbic system, such as the amygdala, insula, cingulate cortex and hippocampus in patients with TTC (30). However, it remains to be elucidated, whether these anatomic abnormalities are pathophysiological factors contributing to the pathogenesis of TTC or the consequence of TTC (30). Furthermore, cerebral MRI imaging of TTC patients has shown a reduced connectivity of both the brain regions of the limbic system and the autonomic nervous system (30).

## Takotsubo Cardiomyopathy and Psychiatric Disorders

In general, prevalence rates of psychiatric and neurological disorders are reported to be high in patients with TTC (2, 5). Also, TTC patients have been found to have higher rates of psychiatric and neurological disorders compared to ACS patients (2). Common predisposing triggering factors of TTC are life events associated with emotional (e.g., panic or anxiety, surprise birthday parties) and physical (e.g., acute respiratory failure or central nervous system conditions) stress (5, 31). More recent data have shown, that emotional triggers are not as common as physical triggers in TTC patients (5). More specific, existing physical triggers of TTC were found to be independent predictors for in-hospital complications (5). Noteworthy, female TTC patients reported more anamnestic emotional triggers prior the occurrence of TTC than male TTC patients, who showed physical triggers prior to the onset of TTC more often (5). Smeijers et al. demonstrated in a small retrospective analysis TTC patients exhibit significant higher levels of depressive symptoms in well-established Patient Health Questionnaire compared to healthy controls (32). Additionally, data derived from the International Takotsubo Registry revealed that 42.3% of studied TTC patients were diagnosed with a psychiatric disorder, whereby 50.0% of these TTC patients had an affective disorder (5). El-Sayed et al. demonstrated within a large retrospective demographic analysis comparing TTC patients with orthopedic and myocardial infarction patients that TTC patients had higher risk for substance abuse (drug and alcohol abuse) (33). Additionally, the intake of medication to treat affective disorders such as selective norepinephrine reuptake inhibitors, serotonin reuptake inhibitors, or benzodiazepines was reported to be more prevalent in TTC patients than in healthy controls (2, 5). Moreover, TTC patients are reported to not have significantly higher general anxiety levels than healthy controls, but higher levels of illness-related anxiety levels (32). Another study showed a high prevalence of diagnosed chronic anxiety disorder prior to the occurrence of TTC (34). Additionally, preadmission anxiety has been found in a case control study to be associated with the occurrence of TTC (35). Remarkably, Summers et al. suggested chronic psychological stress as a risk factor and acute stress as a triggering factor of TTC (34). Psychoneuroendocrinological seen patients with anxiety disorders or depression show increased sympathetic responses to emotional and physical stressors (36). The emotional stress triggering activation of the autonomic nervous system is mediated via two neurohumoral axes: the sympathetic-adrenal-medulla axis with catecholamine release in the adrenal medulla (immediate activation after stressor) and the HPA axis (activation via chronic stressors) with consecutive cortisol release from the adrenal cortex (36). Notably, also low cortisol blood levels have been reported in patients with chronic stress as compensatory mechanism to avoid hypercortisolism (36). Thus, the inhibitory effects of catecholamine release through high cortisol blood levels disappear and which can result in myocardial stunning (36). However, in order to further elucidate the neuroendocrinological mechanisms in TTC, future studies with larger patient cohorts under controlled study surroundings are necessary.

Further, type-D-personality is a controversial debated risk factor of TTC (2). Interestingly, one study revealed pre-existing psychiatric illness is related with an increased risk of TTC, but not an increased 30 day or long-term mortality (37).

## Diagnostic of Takotsubo Cardiomyopathy

The most important clinical tool in diagnosing TTC appears to be an accurate anamnesis of emotional and physical events prior to the onset of the patient's symptoms, if possible. Additionally, assessment of the patient's medical history, particularly of preexisting psychiatric and neurological diseases, is fundamental in the diagnostical workflow of TTC. As clinical presentation of ACS and TTC patients is similar, firstly blood levels of cardiac enzymes are obtained. Commonly, troponin as marker of cardiomyocytes necrosis is elevated, whereas the creatine kinase is usually only slightly elevated in TTC patients (8). High troponin values were shown to be a predictor of a worse in-hospital outcome, because of e.g., the occurrence of malign arrhythmias (8). However, there are patients where troponin is either slightly or not elevated, which led to the term of disproportionately troponin elevation if compared to the seen wall motion abnormalities in TTC patients (6). In general, peak values of troponin blood levels are lower than in patients with ACS (8). An important clinical marker of TTC is elevation of N-terminal prohormone of brain natriuretic peptide (NT-proBNP), which has been shown to be associated directly with the degree of increased blood concentration of catecholamines as a marker of sympathetic overreaction and the severity of left ventricular dysfunction with associated systemic complications, such as pulmonary edema (38). ECG is routinely performed mainly to rule out acute coronary syndrome and myocarditis as differential diagnosis of TTC. Moreover, >95% of TTC patients show ECG abnormalities during the acute phase (3).

## Electrocardiogram

The electrocardiogram (ECG) can be either completely unremarkable or shows ST-segment elevations or ST-segment depression in leads II, III, aVF, aVR, and V5 to V6 (2). Further, T wave inversions suggestive for cardiac repolarization abnormalities can occur in ECGs of TTC patients (39). Prolongation of QT-Intervals (>500 ms) indicate a higher risk for the occurrence of malign and potential life-threatening arrhythmias, such as torsades de pointes and ventricular fibrillation (3, 40). Consequently, close monitoring of ECG and haemodynamic parameters is recommended in TTC patients. Interestingly, recent clinical studies focused on QT prolongation and inflammatory processes in TTC patients. Song et al. have found significant higher levels of c-reactive protein in TTC patients presenting with QT prolongation in a retrospective analysis (40). Perazzolo et al. correlated the pathophysiological ECG changes in TTC patients with myocardial changes in T2-weighted signal cardiac MRI (41). In this study a correlation of apicobasal gradient of myocardial edema and dynamic T wave inversions and QT prolongation, indicating a dispersion of repolarization between apical and basal myocardial regions have been described (41). Elevated catecholamine blood concentrations are postulated as mutual pathophysiological element in TTC and SAH patients. Over the last decades various studies revealed a possible link between local cardiac sympathetic disruptions and reversible T-wave changes in ECG and is together seen with QTc-prolongation (42).

To differentiate TTC patients with ST-Elevations in ECG from ACS patients and also patients with non ST-Elevations, which are hemodynamically unstable and suspected TTC (typical wall motion abnormalities in TTE) patients need to undergo urgent coronary angiography to exclude relevant stenosis of the coronary arteries (8). In 2018 the InterTAK Diagnostic Score as part of the International expert consensus document on Takotsubo syndrome was published and suggested to be utilized in patients with no ST-elevations in ECG, but high probability of TTC (2). By use of the InterTAK Diagnostic Score patients with symptoms suggestive for an ACS and TTC patients with no ST-elevations in the ECG are distinguished (Table 2) (8). The InterTAK Diagnostic Score is positive, if ≥70 points are achieved. In these patients TTE is the recommended as next diagnostical step. Patients achieving ≤ 70 points are recommended to undergo coronary angiography with left ventriculography. Hemodynamically stable patients, with visualized TTC typical wall motion abnormalities and positive InterTAK Diagnostic Score are recommended to receive a coronary computed tomography angiography to visualize coronary status (8).

**Table 2:** International Takotsubo diagnostic criteria score (InterTAK diagnostic score).

25 points	Female sex
24 points	Emotional stress
13 points	Physical stress
12 points	No ST-segment depression
11 points	Psychiatric disorders
9 points	Neurological disorders
6 points	QTc-Interval prolongation

*TTC, Takotsubo cardiomyopathy*.

*Modified from Ghandri et al. (8)*.

Prior the publication of the InterTAK Diagnostic Score of TTC the modified Mayo Clinic Criteria of TTC were commonly used to diagnose TTC in clinical routine (Table 3) (43). Another diagnostical definition was released by the European Society of Cardiology extending the modified Mayo Clinic Criteria of TTC (Table 4) (3).

**Table 3:** Modified Mayo Clinic diagnostical criteria of Takotsubo cardiomyopathy.

Transient hypokinesis, akinesis, or dyskinesis in the left ventricular mid segments with or without apical involvement; regional wall motion abnormalities that extend beyond a single epicardial vascular distribution; and frequently, but not always, a stressful trigger. Absence of obstructive coronary disease or angiographic evidence of acute plaque rupture. New ECG abnormalities (ST-segment elevation and/or T-wave inversion) or modest elevation in cardiac troponin. Absence of pheochromocytoma and myocarditis.

*ECG, Electrocardiogram*.

*Modified from Akashi et al. (43)*.

**Table 4:** Diagnostic criteria for Takotsubo cardiomyopathy of the European Society of Cardiology.

Transient regional wall motion abnormalities of LV or RV myocardium which are frequently, but not always, preceded by a stressful trigger (emotional or physical). The regional wall motion abnormalities usually extend beyond a single epicardial vascular distribution, and often result in circumferential dysfunction of the ventricular segments involved. The absence of culprit atherosclerotic coronary artery disease including acute plaque rupture, thrombus formation, and coronary dissection or other pathological conditions to explain the pattern of temporary LV dysfunction observed (e.g., hypertrophic cardiomyopathy, viral myocarditis). New and reversible ECG abnormalities (ST-segment elevation, ST depression, LBBB, T-wave inversion, and/or QTc prolongation) during the acute phase (3 months). Significantly elevated serum natriuretic peptide (BNP or NT-proBNP) during the acute phase. Positive but relatively small elevation in cardiac troponin measured with a conventional assay (i.e., disparity between the troponin level and the amount of dysfunctional myocardium present). Recovery of ventricular systolic function on cardiac imaging at follow-up (3–6 months).

*BNP, B-type natriuretic peptide; ECG, Electrocardiogram; LBBB, Left Bundle Branch Block; LV, left ventricular; NT-proBNP, NT-proB-type Natriuretic Peptide; RV, right ventricular*.

*Modified from Lyon et al. (3)*.

## Therapeutic Strategies of Takotsubo Cardiomyopathy

The International expert consensus document on Takotsubo syndrome provides the most important strategies in treatment of TTC. Hence the clinical presentation of TTC patients is similar to ACS patients, prehospital treatment of TTC is identical to patients with ACS. It is recommended in TTC patients presenting in cardiogenic shock to avoid catecholamine treatment, as their use have shown higher mortality rates in TTC patients, which seems to be consistent with the assumed underlying pathophysiological mechanisms of TTC (8, 44). In the presence of left ventricular outflow tract obstruction after-load reducing medication is contraindicated. TTC Patients with primary pump failure may need mechanical left ventricular assist devices or an establishment of venoarterial-extracorporeal membrane oxygenation. Also, in cases of mild TTC close monitoring of patients in an intensive care unit setting is necessary to detect and treat possible arrhythmias adequately. Long term medication with beta-blockers after discharge from hospital, if not contraindicated, should be evaluated. However, randomized studies with large patients cohorts focusing on long-term treatment of TTC are needed.

## Conclusion

In conclusion, TTC is an acute and reversible cardiac disease, which is associated with acute dysfunction of the central and autonomic nervous system. However, detailed molecular mechanisms need to be further elucidated, as the role of circulating and synaptic catecholamines as part of the autonomic nervous system are in their precise pathophysiological role unclear. Beside psychiatric disorders neurological disorders, especially acute neurological disorders have been shown to be associated with the occurrence of TTC. Cardiac enzymes are elevated in most TTC cases. Ultimately, every patient with suspected TTC needs visualization of myocardial wall motion in either TTE and/or coronary catheterization with left ventriculography depending on patient's hemodynamic stability. Non-specific ECG changes in TTC patients are reported, however the initial ECG can show either ST-segment elevations or ST-segment depressions, as well as negative T-waves and QTc-Interval prolongation in the initial ECG Acute treatment of TTC depends on patient's vitals and the occurrence of possible complications, like left ventricular outflow tract obstruction or wall ruptures. In most cases close monitoring for cardiac arrhythmia and symptomatic therapy is sufficient. In order to understand TTC in more detail and to develop specific cardiac diagnostical tools and therapeutic strategies both molecular and clinical research need to be performed in future. However, the recently published International expert consensus document on Takotsubo syndrome allows an extensive clinical characterization of TTC and should be used in daily clinical routine to provide excellent patient care.

## Author Contributions

SB conceptualized and drafted the manuscript. DL made substantial intellectual and editing contributions to the manuscript. CS reviewed the manuscript for style and language.

### Conflict of Interest Statement

The authors declare that the research was conducted in the absence of any commercial or financial relationships that could be construed as a potential conflict of interest.
